# Surrogate Decision-Making by Family Caregivers for Hyperthermic Intraperitoneal Chemotherapy in Gastric Cancer: Qualitative Study in a High-Volume Chinese Center

**DOI:** 10.2196/80471

**Published:** 2026-01-19

**Authors:** Zheng-Ke-Ke Tan, Dan-Ni Li, Kui Jia, Wen-Zhen Tang, Xin Chen, Li Yang

**Affiliations:** 1 Nursing Department First Affiliated Hospital of GuangXi Medical University Nanning China; 2 Gastrointestinal Surgery Department First Affiliated Hospital of GuangXi Medical University Nanning China

**Keywords:** hyperthermic intraperitoneal chemotherapy, gastric cancer, family caregivers, surrogate decision-making, qualitative study

## Abstract

**Background:**

Hyperthermic intraperitoneal chemotherapy (HIPEC) has been integrated into the management of gastric cancer (GC) as a combined approach for addressing peritoneal metastasis, serving both prophylactic and therapeutic roles following GC surgery. The pivotal decision regarding HIPEC administration typically arises intraoperatively, creating a complex clinical scenario where family caregivers must act as surrogate decision-makers under substantial time constraints. This decision-making process proves particularly challenging due to limited understanding of the procedure’s risk-benefit profile and long-term outcomes among nonmedical surrogates, challenges often exacerbated by the acute stress of the surgical environment.

**Objective:**

This qualitative study aims to explore how family caregivers of patients with GC navigate the HIPEC decision-making process, specifically examining the facilitators, challenges, and the role of information acquisition that shape the shared decision-making mode.

**Methods:**

This study adopted a qualitative approach using semistructured interviews; 15 family caregivers of patients with GC in a major tertiary hospital in Guangxi Province were selected as research objects through a purposive sampling method. Participants were asked to comment on their experience of surrogate decision-making for the HIPEC process. The Colaizzi 7-step method was used to analyze and summarize the themes.

**Results:**

The mean age of the 15 participants was 39.8 (SD 13.29, range 20-68) years, and all patients were on average aged 56.7 (SD 10.78, range 36-74) years. The relationship to the patient was distributed as follows: 33% (5/15) spouses, 60% (9/15) children, and 6% (1/15) other relatives. Four major themes emerged from the data analysis: (1) shared decision-making participation mode (doctor-led passive decision-making and doctor-family shared decision-making); (2) decision-information sources (decision-making information came from medical-care personnel, decision-making information came from the internet, and decision-making information came from acquaintances); (3) challenges in the decision-making process (financial burden and anticipated therapeutic efficacy); and (4) facilitator in the decision-making process (positive health beliefs and cultural dimensions of perceived responsibility: a Confucian perspective).

**Conclusions:**

HIPEC decision-making by family caregivers of patients with GC was primarily passive decision-making, and many obstacles and facilitators were encountered in the process. Medical staff should share information and encourage and guide family caregivers to participate in the decision-making process through decision assistance or decision support.

## Introduction

Gastric cancer (GC) is 1 of the 5 most common malignant tumors in the world [1], and the cumulative risk of peritoneal metastasis (PM) is nearly 30%. It is the most common form of GC metastasis [2,3]. Systemic chemotherapy drugs have difficulty penetrating the peritoneal barrier; PM treatment is difficult, and the disease progresses rapidly. Once PM occurs, the mortality of patients is very high, which has become the main cause of death in the literature.

Developments have been made in the field of peritoneal tumors over the last decade, and hyperthermic intraperitoneal chemotherapy (HIPEC) is an emerging treatment technique. It heats an infusion-containing chemotherapy drug to the therapeutic temperature and is reinjected into the patient’s abdominal cavity for a certain period of time. The main principle of HIPEC is hyperthermia, chemotherapy, and mechanical scour, which can break through the plasma-peritoneal barrier, remove the residual tissue, and free cancer cells after surgery [4,5]. HIPEC prevents PM based on radical gastrectomy and can also be combined with cytoreductive surgery to treat selected patients with resectable primary or secondary peritoneal malignancy [6,7]. The median survival of patients with advanced GC can reach 18.1 months after HIPEC [8]. However, there is some controversy about the safety and effectiveness of HIPEC. A meta-analysis reported that HIPEC failed to improve the 1-3 year overall survival and gastrointestinal complication rates, but improved long-term overall survival [9]. From the perspective of health literacy popularization, a small percentage of the public has heard of or understood HIPEC, which is a complex operation [10]. Treatment decisions in HIPEC are individualized and require patient and physician involvement. Therefore, the difficulty for patients is to question whether the disease can be successfully treated, as HIPEC is unknown. These concerns and questions can run from decision-making to treatment.

Surrogate decision-makers are more common in intensive care units, ignoring the fact that patients with cancer may also need family caregivers to make surrogate decisions [11,12]. Family caregivers often participate in decision-making, and even become the main decision-maker, when some patients with GC may have adverse psychological conditions, physical discomfort resulting in reduced decision-making ability, or surgeons need to put forward HIPEC-related treatment recommendations according to intraoperative conditions. Although the safety of HIPEC has been proven, it is associated with higher mortality, lower overall treatment morbidity, and reduced quality of life in the short term [13]. People sometimes make different medical decisions for others than they would make for themselves [14]. Family caregivers are usually concerned that surrogate decision-making will influence the patient’s prognosis and lack of HIPEC experience, thus they may fall into decision-making difficulties and conflicts, resulting in possible psychological pressures such as regret, anxiety, contradiction, and unease [11]. Negative emotions will again affect the implementation of decision-making and reduce the satisfaction of patients and caregivers with medical treatment, forming a vicious circle [15].

Previous studies have been conducted to explore the surrogate decision-making process from diagnosis to treatment in patients’ relatives with digestive tract tumors because their lack of understanding of treatment information leads to passive decision-making [16]. HIPEC is a special treatment for gastrointestinal tumors, and most studies focus on prognosis and adverse reactions [13]. Conversely, relatively few studies have focused on the surrogate decision-making experience of HIPEC, which may be due to the underrepresentation of families of patients with GC in HIPEC clinical trials. Different surrogate decision-makers have different views on health, disease, and participation mode of treatment, which are important for whether patients can obtain better treatment plans in time [12].

This study aims to identify the decision-making participation mode of surrogate decision-makers for patients with GC considering HIPEC, particularly their stance on the spectrum of paternalistic, physician-led decision-making and collaborative, shared decision-making, while elucidating the multilevel challenges and facilitators underlying this process. Our results can serve as a reference for formulating targeted decision-support strategies and guiding doctor-patient communication, thereby improving HIPEC decision-making efficiency, safeguarding patients’ physical and mental health, and enhancing patient satisfaction with medical treatment.

## Methods

### Design

An exploratory qualitative study involved semistructured interviews with family caregivers of patients with GC treated with HIPEC who acted as the primary decision-makers. From January to April 2024, family caregivers of patients with GC undergoing abdominal HIPEC after gastrointestinal gland surgery in a major tertiary hospital in Guangxi were selected as the research subjects. We used face-to-face interviews to understand the real experience of family caregivers’ HIPEC decision-making process. This study followed the comprehensive criteria for the reporting of qualitative research guidelines (Multimedia Appendix 1) [17].

### Study Setting

This study was conducted in the Gastrointestinal Surgery Department of the First Affiliated Hospital of Guangxi Medical University, a national key clinical construction specialty, a key medical and health discipline of Guangxi, and the chief unit of the Guangxi Gastric Cancer Alliance, focusing on the diagnosis and treatment of GC. Through the combination of multiple disciplines, the department carries out accelerated rehabilitation surgery for GC and promotes the rapid rehabilitation of patients with GC. The related technology of accelerated rehabilitation surgery is in a leading position in the country. Doctors evaluate the need for HIPEC before or during surgery, and the patient or family decides whether to proceed with HIPEC after surgery. All patients underwent HIPEC within 48 h after closing the abdominal cavity. The perfused temperature was 43 °C, and each operation time was 60-90 minutes.

### Research Team and Interviewer Characteristics

This study’s team comprised 6 health care providers, namely, 3 postgraduate students (ZKKT, DNL, and XC), 2 specialists in gastrointestinal surgery nursing (KJ and WZT), and 1 expert in the field of oncology nursing (LY). All interviews were conducted by 2 primary researchers (ZKKT and DNL), both are female and holding MSc Nursing degrees, with over 3 years of dedicated research and academic training in oncology. Before data collection, the interviewers completed formal training in qualitative methodology, including specialized instruction in interview techniques, probing strategies, and ethical considerations. To minimize potential bias, neither researcher had established clinical relationships with any of the participants’ families. This absence of prior therapeutic engagement was explicitly disclosed to all participants at the commencement of each interview session [17]. This study was supervised by KJ, a professor of nursing with extensive research experience in gastrointestinal oncology. The project was led by author LY, Director of Nursing at a major tertiary hospital in Southern China.

### Recruitment and Sample

The purposive sampling method was used to recruit the participants. Participants were surrogates of adult patients with GC. We approached surrogates of nondecisional patients who required or were expected to require HIPEC after surgery. To be eligible to participate, surrogates needed to be at least 18 years of age, be fluent in the Chinese language, have clear awareness with sufficient communication skills, and self-identify as a primary decision-maker for the patient. Exclusion criteria included extraperitoneal disease, ascites >500 mL, physiologic inability to undergo HIPEC [5], surrogates of patients with recurrent disease, or those who were not involved in the treatment decision. This approach ensured that all participants had direct experience with the phenomenon of interest. Potential eligible surrogates were screened through electronic health records. After the patient completed the HIPEC treatment, the primary nurses of the patients in charge invited the respondent and confirmed eligibility. Participants were informed that their participation was entirely voluntary and that they could opt out at any time. All participant records were kept confidential.

Sample size was based on data saturation, and the calculation and evaluation methods included 3 elements, namely, base size, run length, and new information threshold [18]. Recruitment was stopped when the 2 researchers considered that the content was saturated and without new aspects emerging. Base size as denominator, the run length was the number of interviews within which we looked for and calculated new information. The new information threshold represents the proportion of new information. We will prospectively calculate saturation using a base size of 4 interviews and a run length of 2 interviews. We have selected a new information threshold of ≤5% to indicate that we have reached adequate saturation.

### Data Collection

Interviews were conducted in a quiet hospital room, a doctor’s office, or a classroom. The interviewers first introduced the purpose, significance, and content of the interview to the interviewees and obtained their consent. Before the interview, interviewees were required to fill in a self-made questionnaire, which included demographic variables (age, gender, education level, occupation, relationship between family caregivers, and patients) of patients and their families, as well as disease-related information (HIPEC protocol and tumor stage) of patients.

**Figure 1:**
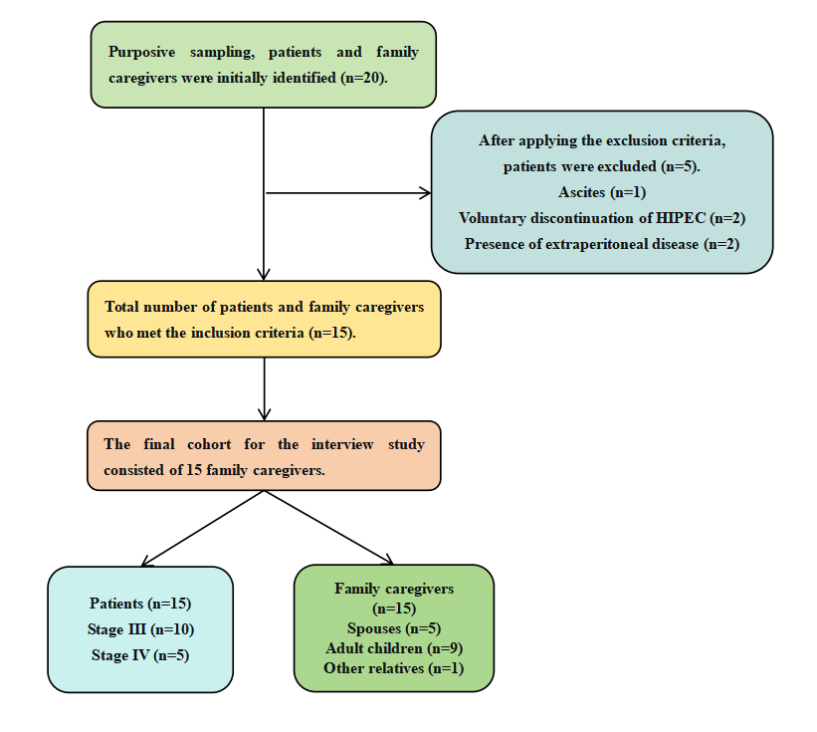
Study enrollment diagram. HIPEC: hyperthermic intraperitoneal chemotherapy.

They conducted a one-to-one semistructured interview with the interviewees according to the final guide of the interview (Textbox 1), which was developed and tested in 2 preinterviews whose interview data were not included in the data analysis. During the process, they paid attention to observe and record the changes in the nonverbal communication of the interviewees. They were encouraged to express their real thoughts and ask questions. All interviews were audio recorded, transcribed verbatim, and compared with digital audio recordings to ensure the accuracy of the content [17]. Unclear or doubtful contents expressed by the interviewees were confirmed through retelling, questioning, and other ways. Induction, intervention, or judgment was prohibited. The interview duration was limited to approximately 30 minutes per participant. A recruitment diagram appears in Figure 1.

**Qualitative interview guide.**

- I'd like to start by asking how did you decide to receive hyperthermic intraperitoneal chemotherapy (HIPEC) on behalf of the patient.
- Can you recall the process of treatment decision?
- What do you know about HIPEC?
- What made you decide to receive HIPEC?
- What concerns did you have during the decision-making process?
- Is there anything you'd like to know or want the medical staff to help you with?

### Data Analysis

The audio recordings from each interview were transcribed verbatim. Within 24 h after the interview, 2 researchers (ZKKT and DNL) independently organized the interview data, transcribed it into text, coded transcripts, and analyzed the data with the Colaizzi 7-step analysis method [19]: The interview data were read repeatedly to deeply understand the content information; code recurring, meaningful statements; form a categorized coded view; write a detailed description; have sublimation view as the theme; and then to return the written text to the interviewee for verification. The 2 researchers were fully immersed in the interview content, independently reading and coding in detail. It was summarized after completion, and if any objection was raised, they were discussed and determined within the team (ZKKT, DNL, XC, and WZT). During this process, the research team adopted triangulation, randomly selected 20% transcripts and codes, and then submitted them to the noninterviewers for reviewing and improving the codes [20]. Then, regular meetings were held within the team to generate initial themes (ZKKT, DNL, XC, and WZT).

### Ethical Considerations

This study was approved by the Ethics Committee of the First Affiliated Hospital of Guangxi Medical University (2024-E055-01). Before the interview, the interviewer introduced the purpose, significance, and content of the research to the subjects. All data were used only for research purposes. All participants signed informed consent forms after expressing their willingness to participate. During the interview, participants were free to interrupt the experiment if they chose to do so. Immediately following data collection, the interviewer engaged all participants in a standardized debriefing process to disclose the sham trial. Participants were not provided compensation for participation. All personal identifiers of the participants were removed before analysis to ensure privacy. This study strictly adheres to Beauchamp and Childress’s Four Principles of Biomedical Ethics [21] to establish the theoretical framework for our ethical approach, recognizing that decision-making during a patient’s surgical procedure inherently precludes the preservation of their decision-making capacity.

## Results

### Patient and Surrogate Decision-Maker Characteristics

The analysis sample for data saturation included 15 patients and their surrogate decision-makers. All participants were on average 39.8 (SD 13.29, range 20-68) years of age at the time of interview. The majority were male (11/15, 73%), and 27% (4/15) were female. The relationship to the patient was distributed as follows: 33% (5/15) spouses, 60% (9/15) children, and 6% (1/15) other relatives. All patients were on average 56.7 (SD 10.78, range 36-74) years of age at the time of interview. The demographic and clinical characteristics of these participants and patients are presented in Table 1.

**Table 1:** Characteristics of participants.

Number	Surrogate decision-maker						Patient					
	Gender	Age (years)	Education	Occupation	Relationship with patients	Gender		Age (years)	Medical insurance	HIPEC^a^, n	Stage	Chemotherapeutics drugs
1	Male	43	Middle school	Farmer	Husband	Female		46	Medical insurance for residents	3	Ⅲ^b^	Lobaplatin
2	Female	36	Junior college	Office clerk	Wife	Male		36	Medical insurance for residents	3	Ⅲ	Docetaxel
3	Male	20	College	Student	Son	Male		53	No medical insurance	5	Ⅳ^c^	Lobaplatin
4	Male	55	Middle school	Farmer	Husband	Female		51	Medical insurance for residents	3	Ⅲ	Oxaliplatin
5	Male	45	Master	Construction industry manager	Son	Female		67	Medical insurance for residents	3	Ⅳ	Lobaplatin
6	Male	32	College	Office clerk	Son	Male		60	Medical insurance for residents	3	Ⅳ	Docetaxel
7	Male	33	College	Civil servant	Son	Male		60	Medical insurance for residents	2	Ⅲ	Oxaliplatin
8	Female	45	Primary school	Farmer	Wife	Male		47	Medical insurance for residents	3	Ⅳ	Docetaxel
9	Male	26	Junior college	Office clerk	Son	Male		54	Medical insurance for residents	1	Ⅲ	Docetaxel
10	Female	49	College	Civil servant	Daughter	Male		74	Medical insurance for urban employees	3	Ⅲ	Docetaxel
11	Male	51	High school	Worker	Son	Male		73	Medical insurance for residents	3	Ⅲ	Docetaxel
12	Male	45	Junior college	Individual	Full brother	Male		51	Medical insurance for residents	3	Ⅲ	Lobaplatin
13	Male	26	High school	Farmer	Son	Male		58	Medical insurance for residents	1	Ⅳ	Oxaliplatin
14	Female	24	Junior college	Individual	Daughter	Male		51	Medical insurance for residents	3	Ⅲ	Oxaliplatin
15	Male	68	Junior college	Retiree	Husband	Female		70	Medical insurance for urban employees	1	Ⅲ	Docetaxel

^a^HIPEC: hyperthermic intraperitoneal chemotherapy.

^b^III: stage III gastric cancer is categorized as locally advanced disease. In this stage, the primary tumor has invaded deeply through the gastric wall (potentially involving the serosa) and has extensively metastasized to the regional lymph nodes.

^c^IV: stage IV represents the most advanced phase of gastric cancer. It is defined by the presence of distant metastasis (M1), regardless of the extent of the primary tumor (Any T) or the involvement of regional lymph nodes (Any N).

### Topic

The researchers identified four themes through their analysis: (1) shared decision-making participation mode, (2) decision-information sources, (3) challenges in the decision-making process, and (4) facilitators in the decision-making process. The themes and subthemes derived from the analysis are shown in Table 2.

**Table 2:** Themes and subthemes.

Themes	Subthemes
Shared decision-making participation mode	Doctor-led passive decision-making Doctor-family sharing decision-making
Decision-information sources	Decision-making information comes from the medical-care personnel Decision-making information comes from the internet Decision-making information comes from acquaintances
Challenges in the decision-making process	The financial burden Anticipated therapeutic efficacy
Facilitator in the decision-making process	Positive health beliefs Cultural dimensions of perceived responsibility: a Confucian perspective

### Theme 1: Shared Decision-Making Participation Mode

#### Overview

The way of shared decision-making was primarily based on the level of information mastery and education of decision-makers.

#### Doctor-Led Passive Decision-Making

Nine surrogate decision-makers expressed a preference for physician-dominated decision-making, indicating that their role was primarily to adhere to the physician’s recommended treatment course.

The adoption of this decision-making approach demonstrates a significant correlation with educational attainment. Surrogates with limited formal education exhibited a substantial knowledge gap regarding HIPEC compared to clinical professionals, while simultaneously expressing stronger deference to hierarchical medical authority.

All of us live in rural areas. I, my wife, my younger brother and my younger sister all have only a middle school education. They seldom go to the hospital for medical treatment. I had never heard of HIPEC and had to rely on the doctor to make the decision. I'm at a loss right now.

Number 4, aged 55 years, farmer, husband of patient

I never considered challenging the doctor's decision. Since the treatment method they proposed was effective, I followed the expert's advice. After all, he was respected authorities whose opinions are widely accepted.

Number 13, aged 26 years, farmer, son of patient

From a cognitive perspective, many surrogates reported experiencing significant feelings of inadequacy and information overload. When participants acquired information but demonstrated either an unwillingness to engage in multisource verification or an inability to synthesize information from disparate channels, they consistently reverted to physician-dependent decision-making as a default strategy.

Several doctors came and told me a lot of information. I managed to understand a little bit, but it was too complicated. Without knowledge of HIPEC, the patient was lying on the bed, unable to move, unable to make decisions. We do what the doctor recommends completely at this time.

Number 11, aged 50 years, worker, son of patient

Although the doctor is obligated to inform us of this information, I don't think I can fully understand it. I don't worry about whether I can understand it or not. All I need to know is that the doctor's decision is correct, and I just need to follow his decision.

Number 1, aged 43 years, farmer, husband of patient

#### Doctor-Family Shared Decision-Making

Six surrogate decision-makers combined information obtained from various sources. Family caregivers who engaged in doctor-family shared decision-making typically possessed higher educational attainment, demonstrating not only comprehension of complex HIPEC information but also the capacity to critically evaluate disparate information sources, including both physician-provided guidance and internet-acquired content.

At the beginning, after being informed of HIPEC by the clinical doctor, first checked the Internet, but also asked acquaintances, to understand the thermal perfusion technology can prevent metastasis, avoid risk effect, we decided to carry out HIPEC.

Number 5, aged 45 years, construction-industry manager, son of patient

On the premise of knowing a lot about the principle, knowledge and effect of thermoperfusion, I took the initiative to choose the best hospital recognized for surgery and HIPEC.

Number 6, aged 32 years, office clerk, son of patient

These surrogate decision-makers did not passively await information but rather demonstrated a proactive communication stance, exemplified by actively seeking clarifications during consultations and engaging in iterative questioning with physicians. They perceived clinicians as collaborative partners in problem-solving rather than unidirectional authorities.

After consulting with acquaintances, I repeatedly confirmed with the doctor whether changing the treatment plan could cure gastric cancer and also determined whether the quality of life would change in the future.

Number 5, aged 45 years, construction-industry manager, son of patient

After I came to this hospital now, I will also ask the doctor to check the information I found online. Even though they urged me, I couldn't make up my mind easily.

Number 6, aged 32 years, office clerk, son of patient

### Theme 2: Decision-Information Sources

#### Decision-Making Information Came From Medical Care Personnel

All participants indicated that medical personnel were the initial source of information for their HIPEC decision-making. As a new therapeutic modality, HIPEC has a low popularity among the public, with less access, and obtaining relevant information initially in daily life was difficult for patients and their families.

I've never heard of HIPEC, and I don't know how it works. The first time I heard HIPEC, it was introduced by the surgeon.

Number 2, aged 36 years, office clerk, wife of patient

I usually read disease-related information on social platforms, but rarely mention HIPEC, and only know it from doctors.

Number 10, aged 49 years, civil servant, daughter of patient

The main source of HIPEC information for 10 (10/15, 66.6%) surrogate decision-makers of patients was medical staff. A total of 6 surrogate decision-makers did not subsequently seek information in other ways, so this became their only access to information. In particular, older decision-makers living in rural areas lack the competence and access to information.

We rural people do not know this, only through the doctor's recommendation, and do not look up other information.

Number 1, aged 43 years, farmer, husband of patient

Born in a rural area and an elderly person, I rarely use a smartphone, so I only know about it from my doctor.

Number 4, aged 55 years, farmer, husband of patient

#### Decision-Making Information Came From the Internet

Some surrogate decision-makers indicated that they actively sought information and confirmation through internet channels, these being by those with higher education or high internet usage. The primary information sought by participants concerned 3 key areas: the mechanism of HIPEC treatment, its efficacy in GC, and potential adverse effects.

After the doctor first told me HIPEC, I searched on the Internet myself to simply understand what therapeutic effect it has on GC. I looked it up through wechat public account and Baidu.

Number 2, aged 36 years, office clerk, wife of patient

I searched for side effects (of HIPEC) through Baidu. There are other doctors on Baidu, they might give me different advice.

Number 7, aged 33 years, civil servant, son of patient

Furthermore, the field of HIPEC is highly specialized, and some of the family caregivers of patients have difficulties understanding it.

But I have no knowledge reserve, even if I find relevant information, I cannot understand.

Number 7, aged 33 years, civil servant, son of patient

#### Decision-Making Information Came From Acquaintances

In addition to seeking information through the internet, 3 decision-makers consulted expert acquaintances. For unknown professional treatment information, patients’ families tended to consult other doctors through trusted relatives and friends. Surrogate decision-makers find it easier to hear the truth from close friends, to obtain better judgment, and ensure information credibility.

I have relatives and friends who are doctors, they asked someone who is an expert in the field of stomach cancer to know if HIPEC can help my mother's condition.

Number 5, aged 45 years, construction-industry manager, son of patient

I asked the doctor at our local hospital about the effect of HIPEC and whether it was harmful to the patient's health. After all, it was the first time I heard about this treatment, and I was still a little afraid.

Number 6, aged 32 years, office clerk, son of patient

### Theme 3: Challenges in the Decision-Making Process

#### Financial Burden

An overarching theme from the interviews was that the significant economic pressure associated with postoperative HIPEC presented a major decisional impediment for nearly all participants. This financial strain was frequently cited as a direct barrier to completing the full course of recommended therapy.

As ordinary people, the economic pressure is relatively large, the thermoperfusion drainage tube is completely self-funded with thousands of yuan each time. I hope the society can be helpful in this respect.

Number 11, aged 51 years, worker, son of patient

The price is very expensive and the medical insurance is not reimbursed, our average family salary is not high. what if the treatment results are not proportional to our efforts?

Number 9, aged 26 years, office clerk, son of patient

#### Anticipated Therapeutic Efficacy

First, a notable uncertainty was noted among some participants concerning the expected benefits of HIPEC, particularly pertaining to the therapeutic protocol and its effectiveness in preventing metastasis and recurrence. This ambiguity was rooted in their nonprofessional background, which consequently hindered their comprehension of the proposed HIPEC regimen and led to profound decisional uncertainty.

The doctor and I have discussed whether to do it three times or four times before, but we still don't know what effect the number of times has on the efficacy, and we are not sure whether the patient can complete the radical cure or prevent metastasis after completing HIPEC, This makes me quite unsure whether I really need it.

Number 2, aged 36 years, office clerk, wife of patient

I mainly want to know what effect can be achieved by doing three times, although the doctor always said that the effect is not guaranteed, I hope you can take the initiative to give us a clear answer.

Number 5, aged 45 years, construction-industry manager, son of patient

Second, some participants, after an in-depth understanding of HIPEC, were concerned that the new technology in chemotherapy had unknown risks of damaging health or aggravating conditions.

HIPEC is also a form of chemotherapy that kills cancer cells and seems to kill healthy cells, raising concerns that patients may suffer weakened immunity and other after-effects.

Number 2, aged 36 years, office clerk, wife of patient

I'm still worried about any adverse reactions or accidents with this new technology.

Number 10, aged 49 years, civil servant, daughter of patient

### Theme 4: Facilitator in the Decision-Making Process

#### Positive Health Beliefs

The participants showed a desire to assist patients in their recovery and were the most important facilitators in decision-making. Such participants indicated that they would be more willing to consent to HIPEC, particularly for patients with GC at high risk of PM. They perceived the procedure as offering a promising therapeutic approach, citing its potential to prevent and treat metastatic dissemination, improve survival outcomes, and maintain a few side effects profile. Surrogate decision-makers considered that health was more important than wealth.

I know that the patient is not in good condition now. As long as the curative effect is good, the treatment should be treated, do not think about the problem of money, health is the most important.

Number 14, aged 24 years, individual, daughter of patient

Health is the most important thing. I understand that HIPEC as early as possible after surgery can more thoroughly identify cancer cells, and then prolong life. As long as there is a effective treatment, we must actively coordinate.

Number 6, aged 32 years, office clerk, son of patient

#### Cultural Dimensions of Perceived Responsibility: Confucian Perspective

A total of 60% (9/15) of surrogate decision-makers were children of patients who had the Chinese Confucian cultural values that maintained the integrity of the family and honored their parents. Having a parent with cancer triggered feelings of guilt, and participants felt it was their responsibility to help patients choose the best treatment. They were concerned that making the decision to forgo HIPEC would lead to adverse health outcomes for their parent.

My dad's situation of cancer was bad, but my parents have worked so hard for us since childhood, and as long as there is any hope, I want to try.

Number 10, aged 49 years, civil servant, daughter of patient

My father knew that the cost of HIPEC was very expensive in a huff, and he wanted to give up the treatment. But he had worked hard all his life, and I, as a son, certainly would not give up the treatment easily. Now we comfort him every day and advise him to actively cooperate.

Number 9, aged 26 years, office clerk, son of patient

## Discussion

### Principal Findings

In this qualitative study, 15 family caregivers of patients with GC as surrogate decision-makers were preliminarily studied in China. Applying thematic analysis to those that met our study inclusion criteria, we identified 4 themes, including the mode, facilitators, challenges, and decision-information sources of surrogate HIPEC decision-making.

Our findings identified 2 predominant modes of shared decision-making among HIPEC surrogate decision-makers. Those with lower educational attainment demonstrated reduced health literacy and stronger deference to hierarchical medical authority, making them more likely to rely on a health care provider for decision-making. This appears rooted in their limited capacity to comprehend medical concepts, compounded by inherent information asymmetry that predisposes clinical dominance. Lacking the ability and confidence to navigate this entrenched power structure, they often transferred final decision-making authority to clinicians, thereby mitigating the burden of decisional conflict [22]. Additionally, these decision-makers lack the foundational cognitive skills to assess information reliability and weigh risks against benefits—a finding consistent with Robertson et al [23]. This deficit impedes their ability to process highly specialized information. When confronted with unfamiliar and complex decisions, they frequently experience cognitive and informational overload. Such overload precipitates decisional conflict, characterized by internal contradiction and profound uncertainty, ultimately precluding meaningful participation in either independent or shared decision-making.

Surrogate decision-makers who used diverse information sources were more likely to engage in doctor-family shared decision-making, a finding consistent with Bakke et al [24]. From a personal capacity perspective, their cognitive and educational foundations translated into superior health literacy, enabling them to evaluate the credibility of web-based information and synthesize disparate sources into a coherent cognitive framework. This capacity fostered greater autonomy in assessing treatment efficacy and survival rates through independent verification [25]. Notably, during our interviews, these individuals voluntarily demonstrated their understanding of HIPEC specifics. Regarding behavioral strategies, these surrogates proactively managed information flow to ensure comprehensive understanding and approached decision-making as a process requiring their ultimate accountability. When physicians and patients communicated effectively, they collectively arrived at treatment decisions [26].

The participation of patients and their families in decision-making conformed to the ethical principle of autonomy and should not play a passive decision-making role in this process [27]. Our findings underscore that facilitating effective shared decision-making requires the integrated provision of essential information alongside careful consideration of patient and family values throughout the decision-making continuum. Patient decision AIDS (PDAs) are popular tools in recent years that help patients make the best choices in health care options based on their personal values and goals, based on evidence-based medicine [28]. Online PDA provides treatment options according to the preferences of patients and their families, including related benefits, risks, and diagrams [29]. PDA can realize centralized information and effectively reduce decision-making conflicts in patients with cancer. From the health care team’s perspective, we recommend establishing a dedicated HIPEC decision-support team comprising surgeons and specialized oncology nurses to provide structured decision guidance. This team would be responsible for implementing and systematically evaluating surrogate decision-makers’ comprehension and use of decision aid materials [30]. Whole-process and dynamic decision-making support was provided to improve the health literacy of patients and their families and thus reduce decision-making difficulties and conflicts.

We found that participants lacked the basic knowledge of HIPEC and the ability to identify and understand information. Consistent with the result of Hart et al [31], surrogate decision-makers spent more time understanding decision information, with less information, they often misinterpret and undermine decisions. We found that participants with low education indicated that they were not interested in researching decision information. Xie et al [32] similarly found that caregivers’ desire for information was lower than for decision-making participation. When the family caregivers, as nonprofessionals, were faced with highly specialized information such as HIPEC, they had difficulty understanding it. This inability even destroyed the positive degree of surrogate decision-makers who obtain further information, especially among the less educated [33]. These findings underscore that health care providers must prioritize the dissemination of HIPEC knowledge and enhance health education for patients with GC and their families, as this constitutes a fundamental prerequisite for facilitating informed decision-making. First, implementation should begin by advancing health care providers’ understanding of current HIPEC evidence, enabling them to coherently translate and disseminate updated research insights to surrogates during decision-making consultations. Second, a supportive environment should be created. We recommend that health care institutions implement PDAs containing structured educational modules, which should provide an overview of GC natural history for HIPEC, comparative outcomes of treatment options, and so on. As demonstrated by Jayakumar et al [34], PDAs with professional guidance yield superior decision quality compared to merely distributing educational materials passively. With guidance from health care providers, these tools help laypersons establish fundamental disease and treatment knowledge, creating essential cognitive foundations for decision-making. Furthermore, providing educational videos at hospital admission significantly enhances engagement and health literacy among patients and their families, thereby improving both the decision-making experience and overall satisfaction with care [35].

The participants expressed diverse challenges facing HIPEC decisions. Financial burden emerged as the primary and most formidable challenge during the decision-making process. Consistent with the result of Graves et al [36], the lower-income patients and caregivers with HIPEC were associated with higher rates of decision regret. Some participants said that the single-use perfusion catheters required for HIPEC were a substantial out-of-pocket expense. During initial discussions of HIPEC, clinicians informed patients of the treatment costs but did not sufficiently underscore the substantial financial implications. As a result, families proceeded with the procedure only to discover—after 1 or 2 cycles—that the cumulative expenses far exceeded their anticipated financial burden. Consistent with reports from Western health systems [37], it was compounded in China as HIPEC was currently not reimbursable through national health insurance, representing a significant financial burden borne directly by families. Consequently, most treatment costs must be borne directly by families rather than through social insurance mechanisms. This financial burden compromises the ability of surrogate decision-makers to pursue clinically optimal decisions and develop treatment plans aligned with optimal clinical guidelines [38]. Uninsured patients with PM will be less likely to consider HIPEC or adjuvant chemotherapy, resulting in people with low economic and social status still at a disadvantage in terms of health [37]. Hamilton et al [39] proved that although the cost of HIPEC for patients was higher than that of systemic chemotherapy, the quality and the overall cost-effectiveness are better. Therefore, according to China’s medical conditions, the medical security system should be improved, and the compensation ratio related to HIPEC should be refined to accurately solve the economic burden of patients with GC.

Moreover, the unpredictability of treatment outcomes significantly contributes to this complex decision-making situation. First, most significantly, while the combination of cytoreductive surgery and HIPEC demonstrates efficacy in both treating and preventing peritoneal metastases in advanced GC, this approach remains controversial and continues to evolve within the oncology community [40]. Based on their concern for the patient, participants were concerned about whether a previously unknown treatment can actually improve the cancer situation and inflict new damage to the patient. A joint multicenter study from the United Kingdom and Australia demonstrated significant variations in post-HIPEC survival outcomes and length of hospital stay across different geographic populations [41]. As highlighted by Marano et al [42], even within high-volume specialized institutions, the application of cytoreductive surgery with HIPEC varies substantially in terms of indication, technical execution, and outcome expectations, underscoring the need for clearer communication strategies with patients and their families. Second, our findings indicate that participants who engaged with multiple information channels and possessed some prior understanding of HIPEC experienced particularly pronounced uncertainty, often stemming from encountering conflicting or inconsistent information across these sources. Different from the results of Crijns et al [26], higher health literacy and satisfaction with the decision of decision-makers were associated with sufficient information support for them. We analyzed that the possible cause was cancer information overload, which can be defined as feeling overwhelmed by the amount of cancer-related material in the information environment. Shi et al [43] show that cancer information overload is associated with perceived perception of illness and treatment uncertainty can lead to anxiety, depression, and emotional fatigue, and, in turn, impaired decision-making ability [30,44]. Effective clinician-family communication serves as a fundamental prerequisite for mitigating therapeutic uncertainty among decision-makers, aiming to support their psychological well-being and maintain alignment with treatment goals. In this context, physicians should engage in transparent dialogue with surrogate decision-makers, recognizing that artificial intelligence-based decision aids cannot substitute for essential human interaction [45].

Regarding the facilitator underlying the surrogate decision-making process for HIPEC, participants indicated their choices were informed by positive health perceptions as well as socioculturally informed perceived responsibilities. First, a participant believed that the “hope” brought by HIPEC can restore a healthy life status. Consistent with the results of Boegle et al [46], when decision-makers perceive that HIPEC is meeting their needs, evoked hope and trust, it benefits the adherence to their treatment. To be precise, they are decision-makers who may care more about longer survival and relapse in the future [45]. Second, consistent with Nissen et al [47], family functionality support is also conducive to improving the disease pressure of patients with cancer and their families and to reducing the physical and psychological burden on caregivers. Furthermore, within traditional Chinese cultural norms, adult children serving as surrogate decision-makers typically adhere to the Confucian virtue of filial piety. This ethical framework mandates comprehensive care for elderly parents, and such filial behavior has been associated with mitigated caregiver burden among adult children [48,49]. Seeking aggressive treatment modalities is often viewed as an expression of loyalty and familial responsibility. However, as demonstrated by Wu et al [50], children with strong filial values may feel compelled to pursue intensive treatment options even when facing substantial financial costs, which can paradoxically lead to decision-making conflicts and psychological distress. In summary, we recommend implementing family centric support protocols for individuals considering HIPEC, delivered through multidisciplinary shared decision-making teams that integrate specialized psychotherapists and oncology nurses to provide sustained psychosocial support. This structured approach enhances decision-making quality by systematically leveraging family caregivers’ capacity for optimism as a constructive psychological resource [30,44]. Given the significant role of filial piety in surrogate decision-making, health care providers should cultivate a balanced understanding of Confucian cultural values among adult children and proactively identify decision-making conflicts during clinical consultations.

### Strengths and Limitations

Strengths and limitations of our study warrant discussion. First, our study focuses on the decision-making process of family caregivers of patients with GC, considering HIPEC, an understudied population (to the best of our knowledge). By adopting a qualitative approach, we delve into their lived experiences, addressing a frequently overlooked dimension of patient-centered care. Second, the systematic methodology used enhances the methodological rigor of the inquiry.

Our study has several limitations that should be considered when interpreting the findings. First, our application of a purposive sampling strategy, recruiting participants from a single health care institution in Southern China, means our findings may not be fully representative of all family caregivers of patients with GC considering HIPEC. Second, our cohort consisted predominantly of male participants, reflecting the sociocultural context in China where male family caregivers often assume the role of primary decision-maker. Yet, the framework of culture could also be a strength: this research is contextualized within the framework of Chinese culture, examining the underlying logic and sensitivities of relevant sociocultural norms for the understanding of decision-making processes. Finally, this study was limited by its exclusive focus on the perspectives of surrogate decision-makers. The findings do not incorporate the views of treating clinicians, the patients themselves, or direct observation of clinical interactions, which represent critical avenues for future research to triangulate and enrich our understanding of the decision-making dynamics.

### Future Directions

Regarding future research, we propose several directions. Subsequent studies should aim to disentangle the specific factors involved in both information acquisition and the decision-making process for HIPEC. This includes investigating how individuals receive and process information from various channels and how they navigate psychosocial challenges during decision-making. We further propose large-scale, multicenter studies that use a mixed methods approach engaging multiple stakeholders—including surgeons, oncologists, and patients—to develop an integrated model of HIPEC decision-making. Finally, we recommend that future research intentionally enroll more female surrogate decision-makers to facilitate a gender-based analysis and elucidate its influence on decision-making outcomes.

### Conclusions

This qualitative study explored the real experiences of GC patients’ family caregivers in making decisions about surrogate HIPEC treatment, including the participation mode, decision-making information sources, facilitators, and challenges. Our findings suggested that providing necessary and sufficient information support with regard to HIPEC was the key to achieving shared decision-making by surrogate decision-makers and improving the decision-making experience. Our findings highlighted areas that have not been previously addressed. Health care providers must intensify their efforts to refine the shared decision-making framework for HIPEC and develop culturally sensitive PDA tailored to the sociocultural and health care context. Such instruments are essential to support GC families in achieving optimal surrogate decision-making aligned with patient values and clinical evidence.

## Appendix

Multimedia Appendix 1 Tables of a qualitative interview guide, participant characteristics, and themes and subthemes.

## Abbreviations

GC: gastric cancer
HIPEC: hyperthermic intraperitoneal chemotherapy
PDA: patient decision AIDS
PM: peritoneal metastasis
