# Supplementary material for: Surrogate Decision-Making by Family Caregivers for Hyperthermic Intraperitoneal Chemotherapy in Gastric Cancer: Qualitative Study in a High-Volume Chinese Center
Source: JMIR Cancer. 2026 Jan 19;12:e80471. doi: 10.2196/80471 (PMC12865350; doi:10.2196/80471)
Supplement: Multimedia Appendix 1 [file cancer_v12i1e80471_app1.docx]

**Table S1.** Characteristics of participants.

| Number | Surrogate decision maker | | | | | Patient | | | | | |
| --- | --- | --- | --- | --- | --- | --- | --- | --- | --- | --- | --- |
|  | Gender | Age (years) | Education | Occupation | Relationship with patients | Gender | Age (years) | Medical insurance | HIPEC^a^, n | Stage | Chemotherapeutics drugs |
| 1 | Male | 43 | Middle school | Farmer | Husband | Female | 46 | Medical insurance for residents | 3 | Ⅲ^b^ | Lobaplatin |
| 2 | Female | 36 | Junior college | Office clerk | Wife | Male | 36 | Medical insurance for residents | 3 | Ⅲ | Docetaxel |
| 3 | Male | 20 | College | Student | Son | Male | 53 | No medical insurance | 5 | Ⅳ^c^ | Lobaplatin |
| 4 | Male | 55 | Middle school | Farmer | Husband | Female | 51 | Medical insurance for residents | 3 | Ⅲ | Oxaliplatin |
| 5 | Male | 45 | Master | Constructio-n industry manager | Son | Female | 67 | Medical insurance for residents | 3 | Ⅳ | Lobaplatin |
| 6 | Male | 32 | College | Office clerk | Son | Male | 60 | Medical insurance for residents | 3 | Ⅳ | Docetaxel |
| 7 | Male | 33 | College | Civil servant | Son | Male | 60 | Medical insurance for residents | 2 | Ⅲ | Oxaliplatin |
| 8 | Female | 45 | Primary school | Farmer | Wife | Male | 47 | Medical insurance for residents | 3 | Ⅳ | Docetaxel |
| 9 | Male | 26 | Junior college | Office clerk | Son | Male | 54 | Medical insurance for residents | 1 | Ⅲ | Docetaxel |
| 10 | Female | 49 | College | Civil servant | Daughter | Male | 74 | Medical insurance for urban employees | 3 | Ⅲ | Docetaxel |
| 11 | Male | 51 | High school | Worker | Son | Male | 73 | Medical insurance for residents | 3 | Ⅲ | Docetaxel |
| 12 | Male | 45 | Junior college | Individual | Full brother | Male | 51 | Medical insurance for residents | 3 | Ⅲ | Lobaplatin |
| 13 | Male | 26 | High school | Farmer | Son | Male | 58 | Medical insurance for residents | 1 | Ⅳ | Oxaliplatin |
| 14 | Female | 24 | Junior college | Individual | Daughter | Male | 51 | Medical insurance for residents | 3 | Ⅲ | Oxaliplatin |
| 15 | Male | 68 | Junior college | Retiree | Husband | Female | 70 | Medical insurance for urban employees | 1 | Ⅲ | Docetaxel |

**Table S2.** Themes and subthemes.

| Themes | Subthemes |
| --- | --- |
| 1. Shared decision-making participation mode | - Doctor-led passive decision-making - Doctor-family sharing decision-making |
| 1. Decision-information sources | - Decision-making information comes from the medical-care personnel - Decision-making information comes from the internet - Decision-making information comes from acquaintances |
| 1. Challenges in the decision-making process | - The financial burden - Anticipated therapeutic efficacy |
| 1. Facilitator in the decision-making process | - Positive health beliefs - Cultural dimensions of perceived responsibility: a Confucian perspective |
